# Classification of Alcohol Use Disorders

**Published:** 2003

**Authors:** Deborah Hasin

**Affiliations:** Deborah Hasin, Ph.D., is professor of Clinical Public Health at the Mailman School of Public Health, Division of Epidemiology, Columbia University, and at the College of Physicians and Surgeons, Department of Psychiatry, Columbia University, New York. She is also a research scientist at New York State Psychiatric Institute, New York, New York

**Keywords:** alcohol use disorder classification, diagnostic criteria, reliability (research methods), validity (research methods), construct validity, longitudinal study, predictive validity, factor analysis

## Abstract

Medical conditions and disorders must be carefully defined both for research and for clinical practice. The most widely used definitions for alcohol use disorders are those determined by editions of the Diagnostic and Statistical Manual of Mental Disorders (DSM) of the American Psychiatric Association and the International Classification of Diseases (ICD) of the World Health Organization. Alcoholism treatment studies, human genetics studies, and epidemiology all rely on these definitions, which constitute a near-universal feature of research on alcoholism. Studies consistently show high reliability for DSM–IV and ICD–10 alcohol dependence but lower reliability for alcohol abuse/harmful use. Validity studies indicate that DSM–IV and ICD–10 alcohol dependence diagnoses have good validity, but the validity for alcohol abuse/harmful use is much lower. The hierarchical relationship of alcohol abuse to dependence may contribute to the reliability and validity problems of abuse, an issue likely to be addressed when work begins on DSM–V.

Clear, accurate definitions of medical conditions and disorders are important for research and clinical practice. The most widely used definitions for alcohol use disorders are found in two major classification systems of disease: the Diagnostic and Statistical Manuals of Mental Disorders (DSM) of the American Psychiatric Association (APA), and the International Classification of Diseases (ICD) of the World Health Organization (WHO). Research on treatment, human genetics, and epidemiology relies on these sets of criteria to define alcohol abuse and dependence diagnoses. For example, alcoholism treatment studies often use definitions from the *Diagnostic and Statistical Manual of Mental Disorders, Fourth Edition* (DSM–IV) (APA 1994) to define inclusion criteria for subjects. Genetics studies use definitions from the *Diagnostic and Statistical Manual of Mental Disorders, Third Edition, Revised* (DSM–III–R) (APA 1987); the DSM–IV; or the *International Statistical Classification of Diseases and Related Health Problems, Tenth Revision* (ICD–10) (WHO 1993) to define sets of alcohol-related characteristics (i.e., phenotypes) under study. Epidemiologic research relies on DSM–IV definitions to define the alcohol use disorders enumerated in the general population and in various population subgroups. In addition, clinicians use DSM or ICD definitions as a common language in their communication about patients. DSM and ICD systems also serve an important educational function because they are used as introductory material on alcoholism for students and trainees from a variety of disciplines. As such, the concepts and definitions of DSM and ICD alcohol diagnoses form a unifying framework that underlies research and discussion of alcoholism in the United States and in other countries.

DSM–IV is the most recent edition of the DSM series and is most widely used in the United States. The previous edition, DSM–III–R, is no longer used clinically. This version remains important to researchers, however, because it was the diagnostic basis for several large and ongoing research projects, including the Collaborative Study on the Genetics of Alcoholism (COGA) (Reich et al. 1998). Outside the United States, the ICD–10 is the system more likely to be used (e.g., Conigrave et al. 2002; Lange et al. 2002; Shaikh et al. 2001; Wutzke et al. 2002). This article provides historical background on the development of the current classification systems; describes similarities and differences between DSM–III–R, DSM–IV, and ICD–10; and reviews the evidence for the reliability and validity of the alcohol dependence and abuse diagnoses.

## Historical Background

DSM–IV and ICD–10 define two alcohol use disorders—dependence and abuse. However, classification systems published prior to 1980 included only one disorder, alcoholism. The first editions of the DSM, *Diagnostic and Statistical Manual of Mental Disorders* (APA 1952) and *Diagnostic and Statistical Manual of Mental Disorders, Second Edition* (DSM–II) (APA 1968) did not provide specific diagnostic criteria for alcoholism or any other disorders. Instead, each included brief paragraphs with general descriptions of the disorders. Clinicians found this format easy to use because they could assign diagnoses based on their assessment of the degree of similarity between a patient’s symptoms and the textual descriptions. However, studies conducted in the 1960s showed several unwanted consequences of diagnosing psychiatric disorders without specified diagnostic criteria. Early test–retest reliability studies (see the sidebar on p. 7 for a description of these studies) indicated that the lack of specified diagnostic criteria reduced reliability (Beck et al. 1962; Spitzer et al. 1975). In addition, cross-national psychiatric studies (not specifically focused on alcohol) showed that the absence of specific diagnostic criteria produced inconsistent diagnostic practices, leading to national prevalence statistics that proved to be incorrect when diagnostic practices were standardized and made more specific (Cooper et al. 1972).

**Reliability: General Considerations**

*Definition of Reliability*. The reliability of a procedure is its reproducibility. Low reliability indicates that results of the measure are inconsistent, thus limiting validity and reducing a measure’s ability to show a relationship between what is being measured and other variables, including causes, treatment responses, and consequences. Hence, determining the reliability of diagnostic procedures (or specific criteria or symptoms) is important.

*Clinical vs. Research Assessment*. Clinicians usually evaluate diagnostic criteria in an unstructured way that varies with training, type of treatment facility, and patient characteristics. This may be responsive to patient and institutional needs, but it may not be reliable enough for research, which requires standardized and specific diagnostic procedures to ensure good consistency and reliability (Spitzer et al. 1975). The need for good reliability led to specific diagnostic criteria in DSM–III and subsequent systems as well as to structured diagnostic interviews to assess these criteria.

*Structured Diagnostic Interviews*. The assessment method in most research on alcohol use disorders consists of a structured diagnostic interview that assesses diagnostic criteria with a specified, structured procedure. In any particular study, the reliability of a diagnosis cannot be completely separated from the reliability of the diagnostic interview. However, consistent reliability findings from studies using different diagnostic interviews indicate more general information about the diagnosis.

*Design of Reliability Studies*. Reliability studies involve comparing the agreement between pairs of assessments made on a series of patients. An *inter-rater reliability study* shows the agreement between diagnoses given by an active interviewer and by an observer. The more common and informative design is the *test–retest reliability study*, in which a series of subjects are independently evaluated with a particular diagnostic interview by two or more interviewers. In this type of study, one interviewer completes a diagnostic interview and then a second “blind” interviewer (not present in the original evaluation) administers the same interview without knowing the results of the first interview. The results of the two interviews are then compared.

*Reliability Coefficient*. A reliability coefficient summarizes the agreement level of all pairs of assessments. The most commonly used coefficient is *kappa*, representing the level of agreement beyond what would be expected by chance. The range of kappa values is from 1.0 to −1.0. A kappa of 1.0 indicates that all pairs of raters agreed perfectly on their diagnostic assessments. A kappa of 0.0 indicates agreement no better than chance (like flipping a coin). Negative kappas can occur but are rare. They indicate that raters *disagreed* more often than would be expected by chance. Ordinarily, kappa values of 0.75 and higher are interpreted as indicating excellent reliability. Kappas between 0.60 and 0.74 indicate good reliability, kappas between 0.40 and 0.59 indicate fair reliability, and values of 0.39 or lower indicate poor reliability.

— Deborah Hasin

ReferenceSpitzerRLEndicottJRobinsEClinical criteria for psychiatric diagnosis, DSM–IIIAmerican Journal of Psychiatry132118711921975117265410.1176/ajp.132.11.1187

The reliability studies and the cross-national comparisons spurred the development of more specific classification systems for diagnosing alcoholism and other psychiatric disorders, especially for research purposes. Feighner and colleagues published a landmark set of research diagnostic criteria for alcoholism in 1972 (Feighner et al. 1972) based on observational studies of hospitalized and incarcerated populations (Barchha et al. 1968; Guze et al. 1969) and on published studies of alcoholics, including participants in Alcoholics Anonymous (Jellinek 1960). The Feighner criteria for alcoholism had a relatively complex structure. A long list of symptoms was divided into four categories, and at least one symptom from three of these four categories was required for a definite alcoholism diagnosis. The categories can be seen as indicators of (1) physiological aspects of heavy drinking, (2) loss of control over drinking, (3) antisocial behaviors connected to drinking, and (4) guilt about drinking or impaired interpersonal relationships.

The Research Diagnostic Criteria (RDC) (Spitzer et al. 1978) provided a simpler structure for diagnosing alcoholism. The RDC consisted of a list of 18 possible symptoms, many of them also found in the Feighner criteria. For a definite diagnosis of RDC alcoholism, three of these symptoms were required. To standardize assessment of the RDC criteria, a semistructured diagnostic interview, the Schedule for Affective Disorders and Schizophrenia (SADS) (Endicott and Spitzer 1978) was designed. Test–retest studies of the SADS/RDC evaluation method showed that the reliability of most diagnostic categories was considerably improved compared with unstructured assessments. In particular, the reliability of diagnoses of alcoholism using the SADS/RDC was extremely high (Spitzer et al. 1978). The RDC criteria were used in the first psychiatric epidemiologic survey that classified respondents according to specified diagnostic criteria (Weissman and Myers 1978; Weissman et al. 1980). The RDC criteria also served as the basis for a large multisite longitudinal family study of affective disorders (e.g., Keller et al. 1983, 1984; Rice et al. 1989) that included the criteria for alcoholism. This led to early studies of the natural history of alcoholism with co-occurring major depressive disorder (Hasin et al. 1989, 1996*d*).

The clear success of specific criteria in improving diagnostic reliability led to the inclusion of such criteria across a wide variety of diagnostic categories in the *Diagnostic and Statistical Manual of Mental Disorders, Third Edition (*DSM–III) (APA 1980). DSM–III represented a major development in American psychiatry (Spitzer et al. 1980), as this was the first classification system intended for clinical as well as research use that included specific diagnostic criteria for the major disorders. Most important for alcohol researchers, DSM–III also was the first classification system to present criteria for two alcohol use disorders—abuse and dependence—rather than for alcoholism alone.

The DSM–III criteria for alcohol abuse included virtually every common pathological manifestation of alcoholism except for withdrawal and tolerance. These abuse criteria were organized into two groups: (1) presumed indicators of pathological use and (2) impairment in social or occupational functioning as a result of pathological use, including legal problems and traffic crashes. A duration criterion, at least 1 month of these problems, also was required for the DSM–III alcohol abuse diagnosis. To make a diagnosis of DSM–III alcohol dependence, either pathological use or impairment in social or occupational functioning (as defined in the abuse criteria) was required, plus evidence of tolerance and/or withdrawal. There was no published rationale given for this division into abuse and dependence or for the allocation of symptoms into subcategories.

A small test–retest study (*n* = 39) of the Comprehensive International Diagnostic Interview–Substance Abuse Module (CIDI–SAM) (Cottler et al. 1989) suggested that the DSM–III criteria for alcohol abuse and dependence were reliable. The original version of the Diagnostic Interview Schedule (DIS) (Robins et al. 1981), a fully structured interview designed for nonclinician interviewers, also was designed for DSM–III criteria. When a series of subjects was independently assessed with the DIS by nonclinicians and psychiatrists, psychiatrists confirmed the nonclinician DIS diagnoses of DSM–III alcohol abuse/dependence in almost all cases (Robins et al. 1981). The DSM–III criteria are not in current use. However, the Epidemiologic Catchment Area study (ECA) (Regier et al. 1984), a psychiatric epidemiologic prevalence survey of 20,219 people conducted in the early 1980s, used DIS/DSM–III criteria to diagnose respondents (Regier et al. 1990), including alcohol abuse and dependence (Helzer and Canino 1992; Robins et al. 1984). Ongoing use of the ECA as a source of information on psychiatric epidemiology keeps the DSM–III criteria in current view.

### The Alcohol Dependence Syndrome: Basis of the Present Alcohol Dependence and Abuse Diagnoses

The definitions of alcohol abuse and dependence underwent a marked change between DSM–III (APA 1980) and DSM–III–R (APA 1987). The diagnostic criteria for alcohol abuse and dependence in DSM–III were chosen and structured without reference to published supporting data. In contrast, the criteria for DSM–III–R alcohol dependence were based on a well-defined theoretical rationale (Rounsaville et al. 1986) derived from a published concept of dependence known as the Alcohol Dependence Syndrome (ADS) (Edwards and Gross 1976; WHO 1981). The ADS was conceptualized as an integration of physiological and psychological processes leading to heavy drinking that was increasingly unresponsive to external circumstances or adverse consequences. The combination of physiological and psychological processes was reflected in the text explaining the ADS concept as well as in the criteria given to define it. The ADS clearly differentiated between the dependence process itself and social, legal, and other consequences of heavy drinking, a distinction known as the biaxial concept (Edwards 1986).

## DSM–III–R, DSM–IV, and ICD–10 Alcohol Dependence and Abuse

Edwards’ biaxial concept was reflected in the DSM–III–R criteria for alcohol use disorders by the organization of the criteria for alcohol dependence and abuse. The dependence category was based on the ADS, with three out of nine criteria required (table 1). The alcohol abuse category, which was to be used only among people who did not meet criteria for DSM–III–R dependence, consisted of other types of alcohol-related problems, and only one out of two symptoms was required. Thus, the DSM–III–R dependence category was defined quite broadly, whereas abuse was much narrower. The Substance Disorders Workgroup of DSM–III–R originally intended to include only alcohol dependence in DSM–III–R (Rounsaville et al. 1986). Concerns that some subgroups of patients might be undiagnosed without an additional category, however, led to the inclusion of abuse in the final set of DSM–III–R criteria (APA 1987). DSM–III–R criteria were used in the National Comorbidity Study (Kessler et al. 1994), a U.S. national survey of 8,098 people sponsored by the National Institute of Mental Health (NIMH) and conducted in the early 1990s.

DSM–III–R represented a considerable departure from DSM–III in many respects, including the categorization of alcohol use disorders. Coming only 7 years later, the transition from DSM–III–R to DSM–IV (APA 1994) reflected a much more conservative process; compelling evidence for improvement was required before changes were adopted. Thus, the DSM–IV criteria for alcohol abuse and dependence were similar to the corresponding DSM–III–R criteria. A concern that the DSM–III–R definition of alcohol dependence had been too broad whereas abuse had been defined too narrowly led to some restriction on the DSM–IV dependence category and addition of criteria to the DSM–IV abuse category (table 2). The DSM–IV criteria for alcohol dependence and abuse were used in the U.S. National Longitudinal Alcohol Epidemiologic Survey (NLAES) (Grant 1997) of 42,862 subjects, which was sponsored by NIAAA and conducted in the early 1990s.

At the same time that the American Psychiatric Association was formulating its editions of diagnostic criteria for mental disorders, the World Health Organization was developing a classification system to compile statistics on all causes of illness and death, including those related to alcohol use disorders. The 10th in the series was the *International Statistical Classification of Diseases and Related Health Problems, Tenth Revision* (ICD–10) (WHO 1993). The work on DSM–III–R and DSM–IV influenced the definitions of the psychiatric and alcohol use disorders included in the WHO classification system. Two versions of ICD–10 were published. One version was intended for clinicians’ reference and included only descriptive text of the disorders, similar to DSM and DSM–II. The other was a research version that included specific diagnostic criteria. Efforts were made to coordinate the ICD–10 research criteria with the DSM–IV, although some differences exist. The ADS also was the basis for the ICD–10 alcohol dependence criteria. In the ICD–10, the “secondary” alcohol category is called harmful use (table 2) and allows problem drinking that leads to medical problems to be diagnosed in the absence of dependence.

DSM–III–R, DSM–IV, and ICD–10 cover similar content for dependence (table 1). Whereas the number of criteria differs in each nomenclature (nine in DSM–III–R, seven in DSM–IV, and six in ICD–10), each system requires that at least three criteria be met for the dependence diagnosis to be made. The definitions of dependence in all three systems include tolerance and withdrawal (the physiological indicators of alcohol dependence) among the criteria. However, in contrast to DSM–III, none of the more recent systems *require* these physiologic symptoms for a dependence diagnosis. Other common criteria between the DSM–III–R, DSM–IV, and ICD–10 include a great deal of time spent drinking and neglect of other activities in favor of drinking. DSM–IV and ICD–10 require symptoms to co-occur within a 12-month period, whereas DSM–III–R is less specific about the co-occurrence of symptoms.

The criteria for abuse or harmful use in the DSM–III–R, DSM–IV, and ICD–10 show greater variation than those for dependence. As noted, DSM–III–R abuse criteria are narrower than DSM–IV. Both DSM–III–R and DSM–IV require only one criterion to diagnose abuse, and both include recurrent use of alcohol in physically hazardous situations as one of the abuse criteria. This criterion accounts for slightly less than 50 percent of all abuse cases in the general population (Hasin et al. 1999; Hasin and Paykin 1999a, b). However, the other abuse criteria differ considerably between DSM–III–R and DSM–IV (table 2). In ICD–10, the harmful use criteria consist of mental, physical, or social harm from drinking. An important commonality of abuse/harmful use across the classification systems is that abuse cannot be diagnosed in a person who currently meets criteria for dependence. Hence, abuse is a residual category for current disorders in DSM–III–R, DSM–IV, and ICD–10. DSM–IV and ICD–10 differ somewhat in their treatment of the relationship of abuse and dependence on a lifetime basis. DSM–IV excludes a diagnosis of abuse in a person who was “ever dependent,” whereas ICD–10 does not limit a later diagnosis of harmful use in a person who was formerly dependent.

### Studies on the Reliability of Alcohol Dependence and Abuse

Reliability is an important attribute of a measure of a disorder because it shows its consistency or reproducibility. The sidebar (p. 7) describes common characteristics of reliability studies. Several test–retest studies have been conducted on the reliability of alcohol dependence and abuse/harmful use. Two reliability studies of alcohol use disorders assessed according to DSM–III-R criteria included:

- A multisite test–retest study of patients (*n* = 390) interviewed with the Structured Clinical Interview for DSM–III–R (SCID) by clinician interviewers (Williams et al. 1992)
- Age netics study of alcoholism patients and their relatives interviewed with the Semi-Structured Assessment for the Genetics of Alcoholism (SSAGA) by clinicians, which included a within-center study (*n* = 154) and a cross-center study (*n* = 86) (Bucholz et al. 1994).

Several test–retest studies of alcohol dependence and abuse have been conducted using DSM–IV criteria, including the following:

- A multisite study of patients (*n* = 172) interviewed with the Psychiatric Research Interview for Substance and Mental Disorders (PRISM) by clinician interviewers (Hasin et al. 1996*c*)
- A multisite study of patients (*n* = 296) interviewed with the Alcohol Use Disorders and Associated Disabilities Interview Schedule (AUDADIS) by nonclinician interviewers (Hasin et al. 1997*a*)
- Ast udy of urban household residents (*n* = 473) interviewed with the AUDADIS by nonclinician interviewers (Grant et al. 1995)
- Puerto Rican general care patients (*n* = 169) interviewed with the AUDADIS by nonclinician interviewers (Canino et al. 1999)
- Subjects from Romania (*n* = 149), Australia (*n* = 149), and India (*n* = 197) interviewed with the AUDADIS by nonclinician interviewers (Chatterji et al. 1997).

Almost without exception, the studies indicated excellent reliability for current DSM–IV alcohol dependence or ICD–10 alcohol dependence (i.e., kappas above 70 [for an explanation of kappas, see the sidebar on p. 7]). The reliability of lifetime dependence diagnoses also was good to excellent across these studies. Because these results were consistent across studies using different diagnostic interviews, the results can be considered attributable to the diagnostic criteria themselves rather than to a particular assessment procedure.

Two of these test–retest studies did not differentiate between abuse and dependence (Grant et al. 1995; Williams et al. 1992). However, among those that did, the reliability of abuse/harmful use was notably lower than the reliability of dependence (Bucholz et al. 1994; Canino et al. 1999; Chatterji et al. 1997; Hasin et al. 1996*c*, 1997a). Examination of the abuse/harmful use category or its individual criteria showed that reliability improved when abuse was diagnosed as an independent category (Bucholz et al. 1994; Canino et al. 1999; Chatterji et al. 1997; Hasin et al. 1996*c*) rather than as a residual category as required by DSM–III–R and DSM–IV.

These results suggest that at least some of the reliability problems with alcohol abuse are a result of the hierarchical structure of the category relative to dependence rather than intrinsically unreliable criteria for abuse.

### Studies on the Validity of Alcohol Dependence and Abuse

As described in the sidebar (p. 13), validity research is more complex than reliability research. There are presently no widely accepted biological tests, or “gold standards,” to use as the benchmark of the validity of specific diagnostic measures. To improve the precision of research studies, alcoholism researchers are actively seeking what are called biological endophenotypes. These sets of characteristics would consist of psycho-physiological measures—for example, measurable variations in biochemistry—that indicate the presence, absence, or severity of the disorder. However, because such endophenotypes have not yet been firmly established, validity still is inferred from evidence such as the studies reviewed below. See the sidebar on validity for a description of each type of design and its rationale.

**Validity: General Considerations**

*Definition of Validity*. A more complex concept than reliability, validity refers to the theoretical correctness of a measure (e.g., a set of diagnostic criteria) of a condition that cannot be directly observed.

*Validity and Reliability Research Compared*. Validity research is more complex than reliability research. There is no single validity coefficient, as there is for reliability. Good reliability is a requirement for good validity, but a reliable measure may not indicate the condition of interest. Therefore, studies are needed to show whether a given diagnostic procedure identifies cases that conform to theoretical prediction about the disorder and its relationship to causal variables and consequences, including whether the diagnosis differentiates between people with a disorder and those without it. Several specific validation strategies exist.

*Studies of Natural History/Longitudinal Course*. Longitudinal studies exemplify predictive validity. They are often used to determine the homogeneity of a given diagnostic category (Feighner et al. 1972) or the relationship between two conditions. Consistency of a diagnosis over time suggests validity, whereas high likelihood that one condition will evolve into a specific, different condition at a later time suggests a lack of validity in the distinction between the two disorders.

*Factor and Latent Class Analysis*. Factor analysis involves analyzing the relationships between a set of variables to determine if they appear to be measuring one or more latent variables, called *factors*. A subset of items more related to each other than to other variables in a data set suggests that this subset of items is measuring an underlying construct or condition. The relationship of any item to a particular factor is indicated by its *factor loading*. Factor analysis has been used to address whether the pattern of correlations between items measuring alcohol abuse and dependence indicates one or two distinct factors, and whether alcohol abuse and dependence items load on distinct factors as predicted by the structure implied by DSM–III–R, DSM–IV, or ICD–10. Latent class analysis uses a latent variable with mutually exclusive categories to represent subpopulations in a sample, where subpopulation membership is not observed but inferred from the data. Variables form symptom profiles that are explained by the existence of a small number of mutually exclusive classes.

*Multimethod Comparisons*. The multimethod comparison study of validity involves comparing the results of assessments made by differing methods on a series of subjects. f the assessments agree on diagnosis despite their differences, then they are likely to be measuring a common underlying condition or construct. If the different methods disagree, then there may be validity problems with the condition of interest, or there may be no true underlying condition. For this type of validation, each of the three classification systems (DSM–III–R, DSM–IV, and ICD–10) can be considered a distinct method of assessment, with some overlapping features and nonoverlapping features across the classification systems. In addition, the results of different diagnostic interviews can be compared, because the interview schedules share features with each other and also differ in numerous ways.

*Construct or Concurrent Validity*. If a set of diagnostic criteria is valid, then the diagnoses made by these criteria should show relationships with variables external to the diagnosis in theoretically predicted patterns. An example of this is an increased prevalence of a given disorder in the biological relatives of index cases with the disorder, compared with index cases without the disorder (Feighner et al. 1972).

— Deborah Hasin

ReferenceFeighnerJPRobinsEGuzeSBDiagnostic criteria for use in psychiatric research\265763197210.1001/archpsyc.1972.017501900590115009428

#### Longitudinal (Predictive) Studies

Several studies have been conducted to determine whether the course of alcohol dependence differs from the course of alcohol abuse. This type of study originally was undertaken to examine whether abuse and dependence represent distinct disorders (Hasin et al. 1990). The question about the distinction between abuse and dependence was raised by a common clinical conceptualization of abuse as a prodromal state (i.e., a stage preceding illness onset in which functioning or condition begins to change) or as an early stage of dependence rather than as a condition that is distinct from dependence. If the clinical concept is correct, then a separate abuse category is not justified; however, because people with abuse who remit without evolving into dependence would be unlikely to seek treatment, the perspective from a clinical standpoint might be biased. More appropriate samples would consist of subjects selected from the general population or other nontreatment samples. Five studies have been done based on such samples. All but one used DSM–III–R criteria (the exception being Hasin et al. 1997*d*, which used DSM–IV criteria). These studies include:

- A 4-year followup of a national sample originally assessed in 1969, in which DSM–III–R diagnoses were derived from data collected during the initial assessment (Hasin et al. 1990)
- A 12-year followup study of a national sample of young adults (Grant et al. 2001)
- A 5-year followup study of university-affiliated men (Schuckit et al. 2000)
- A 5-year followup study of patients and their relatives in a genetics study (Schuckit et al. 2001)
- A 1-year followup study of a community sample of heavier-than-average drinkers (Hasin et al. 1997*d*). (A 10-year followup study of this community sample is now under way.)

Taken as a whole, the study results were fairly consistent. Respondents diagnosed with alcohol dependence were likely to remain chronic, though few of the subjects were in treatment. In contrast, respondents diagnosed with abuse were less likely to exhibit symptoms of their disorder at followup and unlikely to have become dependent. Taken as a group, these studies support the validity of the alcohol dependence category as well as the distinction between alcohol dependence and abuse.

#### Factor Analytic and Latent Class Analysis Studies

Several factor analytic studies of alcohol abuse and dependence have been conducted, some using treatment samples or samples predefined as dependent (e.g., Feingold and Rounsaville 1995; Mohan et al. 1995; Morgenstern et al. 1994). These studies showed a single factor with loadings (for more information, see the sidebar on p. 13) for both dependence and abuse items, suggesting that abuse and dependence are manifestations of a single condition. However, the patients in these studies generally were severe cases, and samples designed around the requirement that at least some of the variables be present to be analyzed may produce distorted results.

One study that combined clinical and community samples found either a one- or two-factor solution, depending on which subjects were studied (Nelson et al. 1999). Other studies of national samples were free of potential biases associated with treatment samples. These studies showed two factors generally corresponding to alcohol dependence and abuse (Harford and Muthen 2001; Muthen et al. 1993a,b; Muthen 1996), supporting the validity of the abuse/dependence distinction.

A latent class analysis was used to investigate the assessment of alcoholism in Australian twins (Heath et al. 1994). This study identified five classes of respondents: those with no problems; occasional excessive drinkers; and classes consisting of mild, moderate, or severe alcohol problems that corresponded to levels of alcohol dependence severity. Another latent class analysis of abuse and dependence symptoms was conducted with data from the COGA study, a genetics study of treated alcoholics and their relatives (Bucholz et al. 1996). This analysis found evidence for four classes, generally non-problem drinkers and those with mild, moderate, and severe problems. Little evidence was found in this study for an abuse category.

#### Multimethod Comparisons

As described in the sidebar above, multimethod comparison studies have compared either the results of different classification systems (DSM–III–R, DSM–IV, and ICD–10). The diagnostic instruments include the Alcohol Use Disorders and Associated Disabilities Interview Schedule (AUDADIS) (Grant et al. 1995; Hasin et al. 1997a); the Comprehensive International Diagnostic Interview (CIDI) (WHO 2000); the Structured Clinical Assessment for Neuropsychiatry (SCAN) (Easton et al. 1997; Hesselbrock et al. 1999; Wing et al. 1990); and the Semi-Structured Assessment for the Genetics of Alcoholism (SSAGA) (Bucholz et al. 1994). The AUDADIS and the CIDI are fully structured, meaning that the questions are asked exactly as worded in the interview schedule. The SCAN and SSAGA are semistructured, meaning that suggested probes are provided for each of the items or criteria, but interviewers are expected to use additional probes to verify that responses are correct. Thus, these “methods” share common features but also differ in numerous ways.

The following studies compared DSM–III–R, DSM–IV, and ICD–10 criteria in assessing a series of research subjects. The samples include:

- Substance abuse patients and community residents (*n* = 521) assessed with the CIDI by clinicians (Rounsaville et al. 1993)
- Alcoholism patients and their relatives (*n* = 1,922) assessed with the SSAGA by clinicians (Schuckit et al. 1994)
- A community sample of heavier-than-average drinkers (*n* = 962) assessed with the AUDADIS by nonclinicians (Hasin et al. 1996*a*,*b*)
- A U.S. national survey of household residents (*n* = 42,862) assessed with the AUDADIS by nonclinicians (Grant 1996)
- A10 -country study of patients and nonpatients, a WHO/NIH joint project on the reliability and validity study of substance use disorders (Hasin et al. 1997b) that included a substudy of subjects assessed with the AUDADIS administered by nonclinicians (*n* = 495), a substudy of the CIDI administered by nonclinicians (*n* = 288), and a substudy of the SCAN administered by clinicians (*n* = 287) (Üstün et al. 1997).

Two studies from the WHO/NIH joint project also compared joint assessments of a single classification system on the same patients as made by the AUDADIS, CIDI, and SCAN. These include:

- Ast udy of the ICD–10 criteria (Pull et al. 1997)
- A study of DSM–IV criteria (Cottler et al. 1997).

The results of these studies were very consistent. Cross-method comparisons indicated excellent agreement for alcohol dependence, supporting the validity of this diagnostic category. However, cross-method agreement was consistently lower for abuse/harmful use. Further examination of the abuse/harmful use category or its individual criteria in three of these studies (Cottler et al. 1997; Hasin et al. 1996b; Pull et al. 1997) showed that the reliability of abuse/harmful use improved when diagnosed as an independent category. These results are similar to the results from the reliability studies, suggesting that it is not inherently invalid criteria that are the source of validity problems for abuse/harmful use but rather the residual structure of alcohol abuse/harmful use relative to dependence.

#### Studies of Construct or Concurrent Validity

An important trait used to assess the validity of psychiatric diagnoses, including alcohol use disorders, is family history—that is, a greater prevalence of a disorder among relatives of a person with that disorder than among the relatives of a person not diagnosed with the disorder. Other external validators used to study the validity of alcohol use disorder diagnoses include treatment history for alcohol problems, actual level of alcohol consumption, history of blackouts, and suicidality. The associations of these variables with diagnoses of alcohol dependence and abuse were tested in a community sample of heavier-than-average drinkers assessed with the AUDADIS (Hasin et al. 1997*c*). In this study, the aim was to determine whether diagnoses of alcohol use disorders offered meaningful differentiation from the other heavy drinkers. The study showed that family history of alcohol problems, suicidality, alcohol consumption, blackouts, and treatment for alcohol problems were all associated with diagnoses of DSM–IV alcohol dependence, compared with subjects with no alcohol-related diagnosis. However, only alcoholic blackouts significantly differentiated subjects with a diagnosis of DSM–IV alcohol abuse from nondiagnosed heavy drinkers. This analysis was repeated using data from the NLAES, a large national sample also assessed with the AUDADIS (Hasin and Paykin 1999a). The replication produced similar results for DSM–IV alcohol dependence. With the larger sample, some of the external variables also were associated with DSM–IV alcohol abuse, but the associations were considerably smaller in magnitude than those found for dependence. Thus, construct validation clearly supported the validity of DSM–IV alcohol dependence, whereas weaker, more equivocal evidence was found for DSM–IV alcohol abuse.

A study by Schuckit and Smith (2001) of high-functioning sons of alcoholics and control subjects indicated that both alcohol dependence and abuse could be differentiated from no diagnosis by several external variables, including family history. Cases of dependence and abuse generally were associated with the same external variables, with stronger relationships suggested for dependence than abuse. A study in Puerto Rico used a somewhat different external validation strategy, comparing AUDADIS diagnoses with either psychiatrist diagnosis or best-estimate diagnosis (Canino et al. 1999). The AUDADIS diagnosis of DSM–IV alcohol dependence showed excellent agreement with the psychiatrist or best-estimate diagnosis. Agreement between AUDADIS diagnoses of DSM–IV alcohol abuse and the more clinical diagnostic methods was poor. However, when DSM–IV alcohol abuse was diagnosed nonhierarchically (i.e., independently of dependence), agreement between the lay- and clinically oriented diagnostic methods was excellent. This adds to the consistent picture of validity evidence for dependence, equivocal or poor results for abuse when considered as a residual category, and considerably improved validity evidence for alcohol abuse when considered independently from dependence.

## Conclusion

Over the last several years, considerable evidence has accumulated on the reliability and validity of modern definitions of alcohol dependence and abuse/harmful use. The evidence comes from studies conducted in clinical samples, general population samples, and samples of participants and their relatives in genetics studies, and not only from U.S. samples but also from samples assessed in many countries around the world. The evidence is very consistent regarding the classification of alcohol dependence (Hasin et al. 2003). This diagnosis, as represented in DSM–III–R, DSM–IV, and ICD–10, has consistently been shown to be reliable and valid. Based on the evidence, investigators can use this category in their research with a high degree of confidence. That does not mean that the criteria for dependence cannot be further improved, or that all questions relative to the alcohol dependence category have been answered. For example, for some research purposes (including genetics studies), representing alcohol dependence as a continuous measure rather than a categorical diagnosis may offer more information and statistical power (Bucholz et al. 1996; Hasin et al. 2002; Muthen 1996; Whitfield et al. 1998). However, not all investigators agree on the appropriateness of a continuous or dimensional form for alcohol dependence (Hasin et al. 2003). In addition, several investigators have been interested in identifying subtypes of alcohol dependence. The purpose of such subtypes is to reduce heterogeneity in the diagnostic category so that more can be learned about treatment response (Babor et al. 1992*a*,*b*; Carpenter and Hasin 2001) or causal factors. At present, however, results from clinically or empirically defined subtypes have not been consistent. The dependence and abuse criteria also may differ in their applicability to adolescents (Martin and Winters 1998), a topic in need of further research but not covered in this review. In addition, studies of the physiological specifier of dependence (i.e., manifestations of withdrawal and/or tolerance as part of the dependence syndrome) suggest the need for further research, because withdrawal is a much better predictor of longitudinal course and other severity indicators than is tolerance (Hasin et al. 2000; Schuckit et al. 1998).

The accumulated evidence in support of the alcohol abuse category is far weaker than the evidence for alcohol dependence. The reliability of alcohol abuse when assessed hierarchically (as required in DSM–III–R, DSM–IV, and ICD–10) is often much lower than the reliability of alcohol dependence. Validity evidence for alcohol abuse also is weaker. Though making the diagnosis of alcohol abuse independently from dependence appears to improve its reliability and validity, it is not clear that such a change would be acceptable to different groups of clinicians and researchers. As preparations for work on DSM–V begin, the role and definition of alcohol abuse will be one topic of consideration for the DSM–V work group on substance use disorders (Hasin et al. 2003).

## Figures and Tables

**Table 1:** Alcohol Dependence: DSM–III–R, DSM–IV, and ICD–10 Diagnostic Criteria

	DSM–III–R^1^	DSM–IV 2	ICD–10^3^
**Clustering Criterion**	(A) At least *three* of the following:	(A) A maladaptive pattern of drinking, leading to clinically significant impairment or distress, as manifested by *three* or more of the following occurring at any time in the same 12-month period:	(A) *Three* or more of the following: occurring together for at least 1 month, or if less than 1 month, occurring together repeatedly within a 12-month period:
Tolerance	Marked tolerance—need for markedly increased amounts of alcohol (i.e., at least 50 percent increase) to achieve intoxication; or markedly diminished effect with continued use of the same amount of alcohol	Need for markedly increased amounts of alcohol to achieve intoxication or desired effect; or markedly diminished effect with continued use of the same amount of alcohol	Need for significantly increased amounts of alcohol to achieve intoxication or desired effect; or markedly diminished effect with continued use of the same amount of alcohol
Withdrawal	Characteristic withdrawal symptoms for alcohol Drinking to relieve or avoid withdrawal symptoms	The characteristic withdrawal syndrome for alcohol (or a closely related substance) or drinking to relieve or avoid withdrawal symptoms	Physiological symptoms characteristic of the withdrawal syndrome for alcohol; or use of alcohol (or closely related substance) to relieve or avoid withdrawal symptoms
Impaired Control	Persistent desire or one or more unsuccessful efforts to cut down or control drinking Drinking in larger amounts or over a longer period than intended	Persistent desire or one or more unsuccessful efforts to cut down or control drinking Drinking in larger amounts or over a longer period than intended	Difficulties in controlling drinking in terms of onset, termination, or levels of use; drinking in larger amounts or over a longer period than intended; or a persistent desire or unsuccessful efforts to reduce or control drinking
Neglect of Activities	Important social, occupational, or recreational activities given up or reduced because of drinking	Important social, occupational, or recreational activities given up or reduced because of drinking	Important alternative pleasures or interests given up or reduced because of drinking OR
Time Spent in Alcohol-Related Activity	A great deal of time spent in activities necessary to obtain, to use, or to recover from the effects of drinking	A great deal of time spent in activities necessary to obtain, to use, or to recover from the effects of drinking	A great deal of time spent in activities necessary to obtain, to use, or to recover from the effects of drinking
Inability to Fulfill Roles	Frequent intoxication or withdrawal symptoms when expected to fulfill major role obligations at work, school, or home OR	None	None
Hazardous Use	Drinking in a physically hazardous situation	None	None
Continued Use Despite Problems	Continued drinking despite knowledge of having a persistent or recurring social, psychological, or physical problem that is caused or exacerbated by drinking	Continued drinking despite knowledge of having a persistent or recurrent physical or psychological problem that is likely to be caused or exacerbated by drinking	Persisting with drinking despite clear evidence and knowledge of harmful physical or psychological consequences
Compulsion	None	None	A strong desire or sense of compulsion to drink
**Duration Criterion**	(B) Some symptoms of the disturbance have persisted for at least 1 month or have occurred repeatedly over a longer period of time.	(B) No duration criterion separately specified, but several dependence criteria must occur repeatedly as specified by duration qualifiers associated with criteria (e.g., “persistent,” “continued”).	(B) Three or more of dependence criteria occurring for at least 1 month, or if less than 1 month, occurring together repeatedly within a 12-month period.

SOURCES:

1 American Psychiatric Association (APA) 1987;

2 APA 1994;

3 World Health Organization 1993.

**Table 2:** Alcohol Abuse/Harmful Use: DSM–III–R, DSM–IV, and ICD–10 Diagnostic Criteria

DSM–III–R^1^	DSM–IV 2	ICD–10^3^
(A) A maladaptive pattern of use indicated by at least one of the following: Continued use despite knowledge of having a persistent or recurrent social, occupational, psychological, or physical problem that is caused or exacerbated by use of the psychoactive substance Recurrent use in situations in which use is physically hazardous (e.g., driving while intoxicated) (B) Some symptoms have persisted for at least 1 month, or have occurred repeatedly over a longer period of time (C) Never met criteria for alcohol dependence.	(A) A maladaptive pattern of drinking, leading to clinically significant impairment or distress as manifested by at least one of the following occurring within a 12-month period: Recurrent use of alcohol resulting in a failure to fulfill major role obligations at work, school, or home (e.g., repeated absences or poor work performance related to alcohol use; alcohol-related absences, suspensions, or expulsions from school; neglect of children or household) Recurrent alcohol use in situations in which it is physically hazardous (e.g., driving an automobile or operating a machine when impaired by alcohol use) Recurrent alcohol-related legal problems (e.g., arrests for alcohol-related disorderly conduct) Continued alcohol use despite having persistent or recurrent social or interpersonal problems caused or exacerbated by the effects of alcohol (e.g., arguments with spouse about consequences of intoxication) (B) Never met criteria for alcohol dependence.	(A) [Harmful use] Clear evidence that alcohol use contributed to physical or psychological harm, which may lead to disability/adverse consequences (B) The nature of harm should be clearly identifiable (and specified) (C) The pattern of use has persisted for at least 1 month or has occurred repeatedly within a 12-month period (D) Symptoms do not meet criteria for any other mental or behavioral disorder related to alcohol in the same time period (except for acute intoxication).

SOURCES:

1 American Psychiatric Association (APA) 1987;

2 APA 1994;

3 World Health Organization 1993.
